# Polygenic risk score model for renal cell carcinoma in the Korean population and relationship with lifestyle-associated factors

**DOI:** 10.1186/s12864-024-09974-w

**Published:** 2024-01-10

**Authors:** Joo Young Hong, Jang Hee Han, Seung Hwan Jeong, Cheol Kwak, Hyeon Hoe Kim, Chang Wook Jeong

**Affiliations:** 1 Biomedical Research Institute, Seoul National University Hospital, Seoul, Republic of Korea; 2 Department of Urology, Seoul National University Hospital, Seoul, Republic of Korea; 3 Department of Urology, Seoul National University College of Medicine, Seoul, Republic of Korea; 4 Department of Urology, Myongji Hospital, Gyeonggi-do, Republic of Korea

**Keywords:** Polygenic risk score, Genome-wide association study, Renal cell carcinoma, Korean population, Non-coding variant, Epigenetics, Lifestyle-associated factor

## Abstract

**Background:**

The polygenic risk score (PRS) is used to predict the risk of developing common complex diseases or cancers using genetic markers. Although PRS is used in clinical practice to predict breast cancer risk, it is more accurate for Europeans than for non-Europeans because of the sample size of training genome-wide association studies (GWAS). To address this disparity, we constructed a PRS model for predicting the risk of renal cell carcinoma (RCC) in the Korean population.

**Results:**

Using GWAS analysis, we identified 43 Korean-specific variants and calculated the PRS. Subsequent to plotting receiver operating characteristic (ROC) curves, we selected the 31 best-performing variants to construct an optimal PRS model. The resultant PRS model with 31 variants demonstrated a prediction rate of 77.4%. The pathway analysis indicated that the identified non-coding variants are involved in regulating the expression of genes related to cancer initiation and progression. Notably, favorable lifestyle habits, such as avoiding tobacco and alcohol, mitigated the risk of RCC across PRS strata expressing genetic risk.

**Conclusion:**

A Korean-specific PRS model was established to predict the risk of RCC in the underrepresented Korean population. Our findings suggest that lifestyle-associated factors influencing RCC risk are associated with acquired risk factors indirectly through epigenetic modification, even among individuals in the higher PRS category.

**Supplementary Information:**

The online version contains supplementary material available at 10.1186/s12864-024-09974-w.

## Background

Renal cell carcinoma (RCC) accounts for 90% of kidney cancers and ranks as the seventh most common cancer in the western world; it constitutes approximately 3% of all cancer diagnoses worldwide [1, 2]. In Asia, the incidence of RCC has increased due to the adoption of western lifestyles [3]. Well-known risk factors for RCC include smoking, excessive weight, and hypertension [4, 5]. Additionally, heritability plays a role in certain rare syndromes with predisposed germline mutations in genes such as VHL, FH, and MET [6, 7].

RCC is usually detected incidentally and asymptomatically when diagnosed at an early stage. Early detection through screening is crucial for reducing the morbidity and mortality associated with RCC [8, 9]. Several prediction models based on clinical, biochemical, historical, and lifestyle markers have been developed and validated to predict the diagnosis, grade, stage, and progression of several cancers, including RCC [10]. Similarly, polygenic risk score (PRS) models that use genetic markers to predict the risk of cancers have demonstrated sufficient predictive power, thereby enabling individualized risk management [11, 12].

Genomic architecture and predisposed allele frequencies vary among different ancestries [13]. PRS models utilizing genetic factors predict individual risk more accurately in Europeans compared to non-Europeans, primarily because the majority of genetic discoveries are made within European populations [14]. Europeans represent the largest ethnicity in training genome-wide association studies (GWAS) globally, accounting for 91% of the data, followed by East Asians at 4.9% [15]. Consequently, the accuracy of the Asian-specific PRS is affected by the relatively smaller sample size of genetic studies conducted in Asian populations, thereby lowering precision when estimating the relative risk for each individual [16]. To address this issue, we conducted a GWAS for RCC using genomic data from 992 cases and 3,431 controls in the Korean population.

Favorable lifestyle factors, such as avoiding tobacco and alcohol, following a healthy diet, and engaging in moderate physical activity, serve as an optimal approach to prevent and manage cancers or complex diseases [17]. Numerous studies have revealed that favorable lifestyle factors can mitigate the risk of cancer among individuals with high genetic risk [18–20]. The aim of this study is to identify RCC-susceptible germline variants specific to Koreans, construct a Korean PRS model to assess the risk of developing RCC based on these variants, and evaluate the performance of the PRS model. Furthermore, this study examined whether lifestyle-associated factors interact with the genetic risk expressed as PRS.

## Methods

### Study participants

This study involved 4,991 Korean individuals. We included the cases of 1,120 patients with RCC who were registered in the Seoul National University Prospectively Enrolled Registry for RCC-Nephrectomy (SUPER-RCC-Nx) and had their blood stored in the human biobank [21]. The control group consisted of 3,871 participants from the Ansan/Ansung study of the Korean Genome and Epidemiology Study (KoGES), a population-based prospective cohort study [22]. The baseline survey for the KoGES was conducted in 2001–2002, and a follow-up survey was carried out biennially for 14 years. The participants were selected based on specific criteria, excluding participants diagnosed with any cancer during the baseline survey and those diagnosed with kidney diseases during the follow-up survey. Genotyping was performed using the Korean Chip array, and the same array was used by the Korean National Institute of Health to genotype KoGES samples.

### Korea biobank array (KoreanChip)

KoreanChip comprises more than 833,000 markers, among which 208,000 are functional markers that have been directly genotyped. These data were collected from an extensive dataset of 22 million variants identified in 2,576 sequenced Korean samples. The dataset encompasses 397 whole-genome sequences from the Korean Reference Genome, along with 2,179 whole-exome sequences sourced from various places, such as the T2D-GENES consortium, the Ansung and Ansan study, a cardiovascular disease sequencing study, and the Korean Children and Adolescents Obesity Cohort study [23].

### Quality control (QC)

QC was performed to analyze the samples and variants. Individuals with sexual inconsistencies were excluded from the study based on the principle that the genotype data on the sex of an individual was inconclusive when the homozygosity rate is greater than 0.2 but less than 0.8. Samples with a call rate < 95%, excessive heterogeneity, and genetic relatedness were removed. Single nucleotide polymorphisms (SNPs) with a call rate < 95%, minor allele frequency (MAF) < 5%, and Hardy–Weinberg Equilibrium (HWE) *p*-value < 1.0 × e^− 6^ were also excluded. Batch effect corrections were conducted for cases [24]. The subsequent step involved correcting the batch effects that arose between cases and controls. Importantly, regulations state that results obtained with KoreanChip must be normalized with 5,000 samples registered in the Korean consortium. Consequently, even though cases and controls underwent separate genotyping in different laboratories, they were effectively normalized to each other according to this regulation, which eliminated batch effects. To assess the effect of population substructure, principal component analysis (PCA) was performed before and after merging the datasets of the cases and controls. QC was completed using a combination of R v4.2, Plink v1.9, and bcftools git version 1.17-10 [25].

### Imputation for missing values

Variants that were not directly genotyped or excluded during QC were imputed using Minimac4. Phasing was performed using Eagle v2.4. The ancestry was limited to East Asians with 1000 Genome project phase 3 for the reference genome panel. We filtered the imputed variants with a genotype quality R2 > 0.8 [26]. Post-imputation QC was conducted by applying the exclusion criteria of an MAF < 5% and an HWE *p*-value < 1.0 × e^− 6^. The percentage of imputed data after the post-QC step was 92.72%.

### Statistical analysis for SNP selection

The samples were divided into two: discovery and validation datasets. The validation dataset, including 492 samples (approximately 10% of the total samples), was randomly extracted, whereas the remaining 4,915 samples were retained for the discovery set after undergoing QC. Association testing with RCC was conducted for the discovery dataset. Logistic regression was performed for the GWAS with covariates, including age, sex, body mass index (BMI), hypertension, and smoking. The associated SNPs were filtered using a threshold of 1.0 × e^− 5^ and a false discovery rate (FDR) of 0.05. LD pruning and fine mapping methods were used to identify causal SNPs for predicting RCC risk [27]. Hail 0.2 was used for statistical analysis.

### PRS calculation and optimal performance

The PRS model was constructed using causal SNPs selected from the GWAS results with the validation dataset.

$$PR{S_j} = \sum\limits_{i = 1}^N {{\beta _i} \times \,dosag{e_{ij}}} $$

####   j:

individual

####   i:

variant of individual *j*

####   N:

number of SNPs in the score of individual *j*

where *PRS*_*j*_ is the risk score for individual *j*, *dosage*_*ij*_ is the number of risk alleles for the *i*-th variant, $\beta$_*i*_ is the natural logarithm of the odds ratio [ln(OR)] (or effect size, beta) of the *i*-th variant, and *N* is the number of SNPs in the score [28].

To compare the performance of the PRS models, systematically removing one SNP at a time and starting from the SNP with the highest *p*-value, a receiver operating characteristic (ROC) curve was plotted, and the area under the curve (AUC) was calculated for different numbers of SNPs. The optimal PRS cut-off value was selected at the point of the maximal Youden’s index (sensitivity and specificity) performed using Plink v1.9 and the pROC package in R.

### Association of PRS and lifestyle-associated factors with RCC risk

We selected BMI, smoking status, alcohol intake, and history of hypertension as lifestyle-associated factors related to RCC risk. Although a favorable lifestyle score is commonly calculated by considering obesity, tobacco use, alcohol intake, diet, and physical activity as lifestyle-associated factors, we replaced diet and physical activity with history of hypertension considering our present data and previous studies related to RCC risk [29, 30]. A favorable lifestyle was indicated by BMI < 30 kg/m^2^, no smoking, moderate alcohol intake, and no history of hypertension (see Additional File 1: Table S1). We assigned one point to each favorable lifestyle-associated factor. We categorized combined lifestyle scores into Ideal (favorable lifestyle score of 3 or 4), Intermediate (favorable lifestyle score of 2), and Poor (favorable lifestyle score of 0 or 1). PRS distributions were categorized into Low (0–40%), Intermediate (40–90%), and High (> 90%). We explored the association of favorable lifestyle-associated factors and PRS with RCC risk and further investigated the relationship between lifestyle-associated factors and RCC risk across the strata of PRS using a Cox proportional hazard model.

## Results

### Discovery phase findings

This study included 4,915 Koreans who were divided into two groups to identify risk variants and construct the PRS model. The discovery dataset comprised 992 cases and 3,431 controls, whereas the validation dataset comprised 112 cases and 380 controls. Although RCC can occur at any age, this study focused only on participants aged ≥ 40 years to examine the common effects of these factors on RCC risk (Table 1).

**Table 1:** Study demographics

	All (*N* = 4,915)		Discovery set (*n* = 4,423)		Validation set (*n* = 492)	
Characteristic	Case (*n* = 1,104)	Control (*n* = 3,811)	Case (*n* = 992)	Control (*n* = 3,431)	Case (*n* = 112)	Control (*n* = 380)
Age, years	62.4 (± 10.7)	54.1 (± 8.2)	62.5 (± 10.6)	54.0 (± 8.2)	62.4 (± 11.2)	54.3 (± 8.2)
Sex						
Female	331 (30)	1,783 (47)	298 (30)	1,610 (47)	33 (29)	173 (46)
Male	773 (70)	2,028 (53)	694 (70)	1,821 (53)	79 (71)	207 (54)
BMI, kg/m^2^	25.2 (± 3.4)	24.7 (± 3.0)	25.2 (± 3.4)	24.7 (± 3.0)	25.4 (± 4.0)	24.5 (± 3.0)
Smoking						
No	586 (53)	2,192 (58)	525 (53)	1,982 (58)	61 (54)	210 (55)
Ex	391 (35)	840 (22)	355 (36)	749 (22)	36 (32)	91 (24)
Current	127 (12)	779 (20)	112 (11)	700 (20)	15 (13)	79 (21)
Hypertension						
No	489 (44)	3,169 (83)	444 (45)	2,864 (83)	45 (40)	305 (80)
Hypertension	615 (56)	642 (17)	548 (55)	567 (17)	67 (60)	75 (20)

Mean (± SD): Age, BMI; number (%): Sex, Smoking, Hypertension

Batch effect correction was performed to address the technical variations or non-biological differences between measurements in different sample groups. Substantial correction of the case dataset was performed. Additionally, to assess the effect of the population substructure, PCAs were performed before and after merging the cases and controls. No specific population substructure was observed (see Additional File 1: Figure S1).

**Fig. 1:**
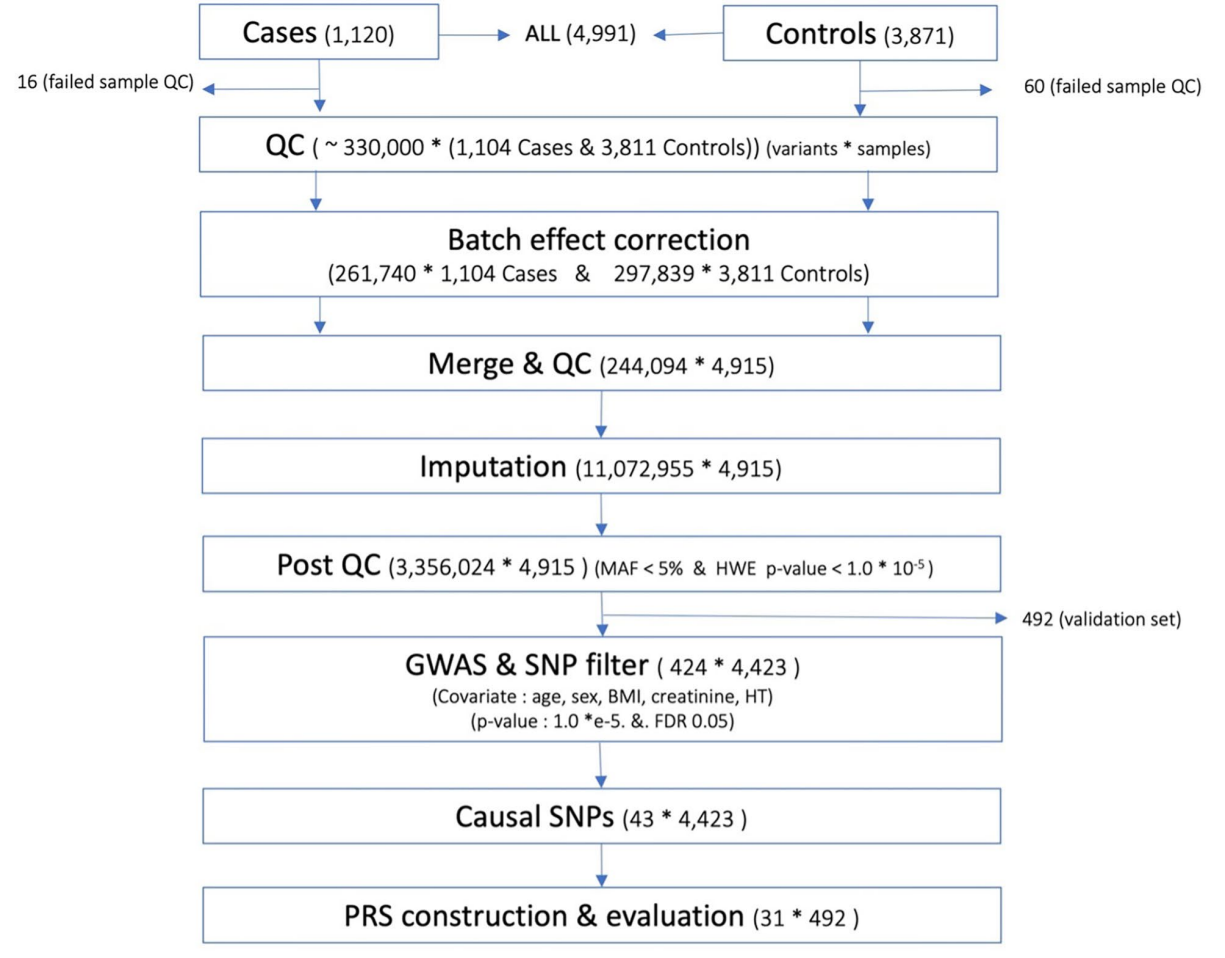
Workflow of the study. This study included patients with RCC from the SNUH and controls from the KoGES. RCC, renal cell carcinoma; SNUH, Seoul National University Hospital; KoGES, Korean Genome and Epidemiology Study; QC, quality control; GWAS, genome-wide association study; SNP, single nucleotide polymorphism; PRS, polygenic risk score; *, multiplication

For the GWAS, logistic regression was used and 424 variants of 4,423 participants were selected [*p* < 1.0 × e^− 5^ and FDR 0.05] (Fig. 1). In the quantile–quantile plot (QQ-plot), the lambda value (λ) was 1.04, indicating no evidence of inflation or acceptable results for the GWAS (see Additional File 1: Figure S2). To identify highly associated causal variants, fine mapping was performed, and 43 out of 424 variants were selected as susceptible loci associated with RCC (see Additional File 1: Table S2).

### Korean PRS construction for RCC risk and biological process of 31 variants

**Fig. 2:**
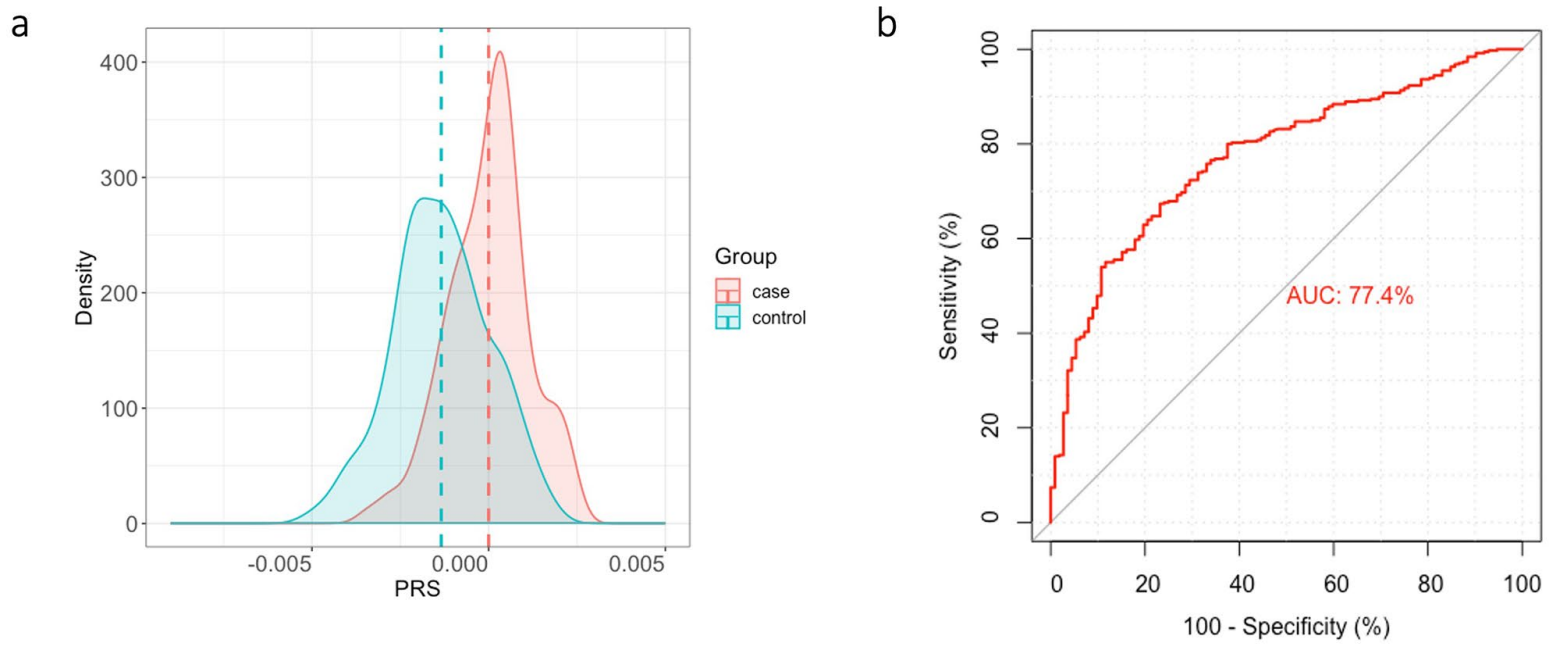
PRS distribution of 31 Korean-specific SNPs and evaluation of PRS performance. The PRS was constructed based on 31 specific SNPs in the Korean population. (**a**) Density plot showing the different distribution of the PRS in cases and controls. (**b**) ROC curve for evaluating PRS performance. SNP, single nucleotide polymorphism; PRS, polygenic risk score; RCC, renal cell carcinoma; ROC, receiver operating characteristic

The Korean-specific PRS model was constructed using 43 SNPs on 492 Korean participants. The maximal AUC value for the PRS model was 77.4% when 31 variants out of 43 were selected (Fig. 2). Although the effect size was not significantly high, the aggregate of the weighted effect size of the 31 SNPs showed a high prediction rate. Of the 31 variants in the PRS model, 15 variants were in the intronic region, 15 in the intergenic region, and 1 downstream (Table 2; see Additional File 1: Figure S3). We annotated these variants with the genes they regulated to investigate whether they were associated with RCC risk. Functions and pathways of the genes regulated by the 15 variants in the intronic region are listed in Table 3.

**Table 2:** SNPs associated with RCC in the Korean population at optimal PRS performance (*n* = 31)

rsID	CHR	POS	REF	ALT	AF	P	OR	IMPUTED	ICGC	GWAS	COSMIC	Function	Gene symbol	cytoBand
rs67756935	3	33,211,952	C	T	0.0958	2.66E-11	4.36E-01	TYPED				intronic	SUSD5	3p22.3
rs6110518	20	15,085,205	G	T	0.08761	1.92E-08	5.04E-01	IMPUTED	ICGC			intronic	MACROD2	20p12.1
rs908237	17	78,914,751	G	A	0.10847	3.01E-08	5.42E-01	IMPUTED				intronic	RPTOR	17q25.3
rs6597341	6	914,841	C	T	0.09013	5.83E-08	5.34E-01	IMPUTED				intergenic	LOC101927691;LINC01622	6p25.3
rs200334091	13	108,890,247	C	T	0.0891	1.79E-07	5.54E-01	IMPUTED				intergenic	ABHD13;TNFSF13B	13q33.3
rs76221875	4	86,702,592	G	A	0.09587	1.81E-07	5.53E-01	IMPUTED	ICGC			intronic	ARHGAP24	4q21.23
rs73149350	7	80,828,288	G	A	0.0721	2.19E-07	5.01E-01	IMPUTED				intergenic	SEMA3C;LOC105369146	7q21.11
rs11868975	17	2,664,626	A	G	0.09587	2.85E-07	5.49E-01	IMPUTED				intergenic	CCDC92B;RAP1GAP2	17p13.3
rs4635969	5	1,308,552	G	A	0.09294	2.98E-07	5.52E-01	TYPED		GWAS		downstream	MIR4457	5p15.33
rs72661905	13	113,568,986	C	T	0.07805	3.46E-07	5.28E-01	TYPED	ICGC			intergenic	ATP11A;MCF2L-AS1	13q34
rs117352285	11	42,410,962	C	T	0.08522	3.94E-07	5.34E-01	IMPUTED				intergenic	LINC02740;HNRNPKP3	11p12
rs11041484	11	7,641,520	G	A	0.08508	4.12E-07	5.45E-01	TYPED		GWAS		intronic	PPFIBP2	11p15.4
rs75488411	19	41,012,097	C	T	0.09048	4.17E-07	5.46E-01	TYPED	ICGC		COSMIC	intronic	SPTBN4	19q13.2
rs11829786	12	57,332,232	C	T	0.07762	4.43E-07	5.28E-01	IMPUTED		GWAS		intergenic	SDR9C7;RDH16	12q13.3
rs57845130	9	17,854,783	G	T	0.07597	5.99E-07	5.23E-01	TYPED				intergenic	SH3GL2;ADAMTSL1	9p22.2
rs35005811	3	14,376,664	T	G	0.24072	6.14E-07	1.41E + 00	IMPUTED	ICGC			intergenic	LSM3;LINC01267	3p25.1
rs78544457	5	121,659,085	G	T	0.10057	7.96E-07	5.84E-01	TYPED				intronic	SNCAIP	5q23.2
rs73584329	9	127,047,016	C	T	0.09449	8.12E-07	5.68E-01	TYPED	ICGC			intronic	NEK6	9q33.3
rs56354798	2	6,594,126	T	A	0.08099	8.53E-07	5.49E-01	IMPUTED				intergenic	LINC01247;LINC01246	2p25.2
rs116907254	14	21,313,268	C	T	0.09977	8.88E-07	5.77E-01	TYPED	ICGC			intergenic	RNASE1;RNASE3	14q11.2
rs12050132	14	73,276,250	A	T	0.50742	9.27E-07	1.34E + 00	IMPUTED	ICGC	GWAS		intronic	DPF3	14q24.2
rs75995217	7	88,849,880	C	G	0.07559	9.60E-07	5.41E-01	IMPUTED				intronic	ZNF804B	7q21.13
rs79192363	5	180,458,802	G	A	0.07741	9.88E-07	5.29E-01	IMPUTED	ICGC			intergenic	BTNL3;BTNL9	5q35.3
rs12813302	12	103,856,908	G	A	0.09099	1.28E-06	5.64E-01	TYPED				intronic	C12orf42	12q23.3
rs13381896	18	25,356,190	T	G	0.90662	1.97E-06	6.31E-01	TYPED				intergenic	LOC105372038;CDH2	18q12.1
rs74588014	9	138,726,580	C	A	0.0938	2.64E-06	5.85E-01	TYPED				intronic	CAMSAP1	9q34.3
rs434919	21	43,333,005	G	T	0.07181	3.14E-06	5.33E-01	IMPUTED	ICGC	GWAS		intronic	C2CD2	21q22.3
rs7973376	12	66,918,796	T	G	0.28683	3.50E-06	7.32E-01	IMPUTED	ICGC			intronic	GRIP1	12q14.3
rs4897331	6	129,866,547	C	T	0.08752	3.71E-06	5.80E-01	TYPED	ICGC			intergenic	LAMA2;ARHGAP18	6q22.33
rs2242402	17	77,918,261	G	A	0.09121	4.25E-06	5.93E-01	TYPED				intronic	TBC1D16	17q25.3
rs17719136	5	147,578,177	G	C	0.08744	4.39E-06	5.88E-01	TYPED				intergenic	SPINK14;SPINK6	5q32

SNP, single nucleotide polymorphism; PRS, polygenic risk score; RCC, renal cell carcinoma; AUC, area under the curve; ROC, receiver operating characteristic; CHR, chromosome; POS, position; REF, reference allele; ALT, alternative allele; AF, allele frequency; P, *p*-value; OR, odds ratio; ICGC, International Cancer Genome Consortium; GWAS, genome-wide association study; COSMIC, Catalog of Somatics in Cancer

**Table 3:** Intronic variants and biological processes (*n* = 14)

Gene	cytoBand	biological process
*SUSD5*	3p22.3	This gene involves the Notch signaling pathway. Notch is the receptor in a highly conserved signaling pathway that is crucial in development and implicated in malignant transformation [42].
*MACROD2*	20p12.1	MACROD2 acts as a haploinsufficient caretaker of the tumor suppressor gene. Loss of function mutations of this gene promote chromosome instability, resulting in cancer evolution [43].
*RPTOR*	17q25.3	The class I phosphoinositide 3-kinase (PI3K)– mechanistic target of rapamycin complex 1(mTORC1) signaling network directs cellular metabolism and growth, which is implicated in diverse pathologies, including cancer, when dysregulated [44].
*ARHGAP24*	4q21.23	As key regulators of cytoskeletal dynamics, Rho GTPases activated by ARHGAP24 coordinate a wide range of cellular processes, including cell cycle progression and cell migration, which enables cancer cells to invade the stroma surrounding the primary tumor [45].
*PPFIBP2*	11p15.4	PPFIBP2 is considered to promote tumor suppressor properties. Germline loss-of-function mutations of PPFIBP2 have been associated with shorter survival in prostate cancer [46].
*SPTBN4*	19q13.2	This gene involves MARK1/MARK3 signaling. This kinase pathway is a central signaling module that participates in physiological and pathological processes like cancer [47].
*SNCAIP*	5q23.2	RNA expression of SNCA and SNCAIP was observed to have a close relationship with medulloblastoma, a brain tumor which has been reported to be related with various tumors [48].
*NEK6*	9q33.3	NEK6 interacts with STAT3, which is an oncogenic transcription factor. It phosphorylates STAT3 on Ser^727^, which is important for transcriptional activation [49].
*DPF3*	14q24.2	This gene is a component of the BAF chromatin remodeling complex (ATP remodeling complex). BAF complex subunits are frequently altered in cancer with up to 20% of human cancers [50].
*ZNF804B*	7q21.13	Differential expression levels of ZNF proteins in different cancer types are regulated by cancer-related miRNA [51].
*CAMSAP1*	9q34.3	CAMSAP1 mutation can activate anti-tumor immunity, mediate tumor cell apoptosis, and improve platinum drug sensitivity [52].
*C2CD2*	21q22.3	C2CD2 mutations were associated with a higher incidence of colorectal adenomas. C2CD2 up-regulation lead to cytosolic Ca^2+^ increase involved in the regulation of apoptosis [53].
*GRIP1*	12q14.3	The PKA-stimulated degradation of GRIP1 leads to changes in the expression of a subset of genes regulated by estrogen receptor-α in MCF-7 breast cancer cells [54].
*TBC1D16*	17q25.3	The TBC domain family is implicated in various cellular events contributing to initiation and development of different cancers [55].

### Relevance of lifestyle-associated factors to RCC risk across PRS strata

**Fig. 3:**
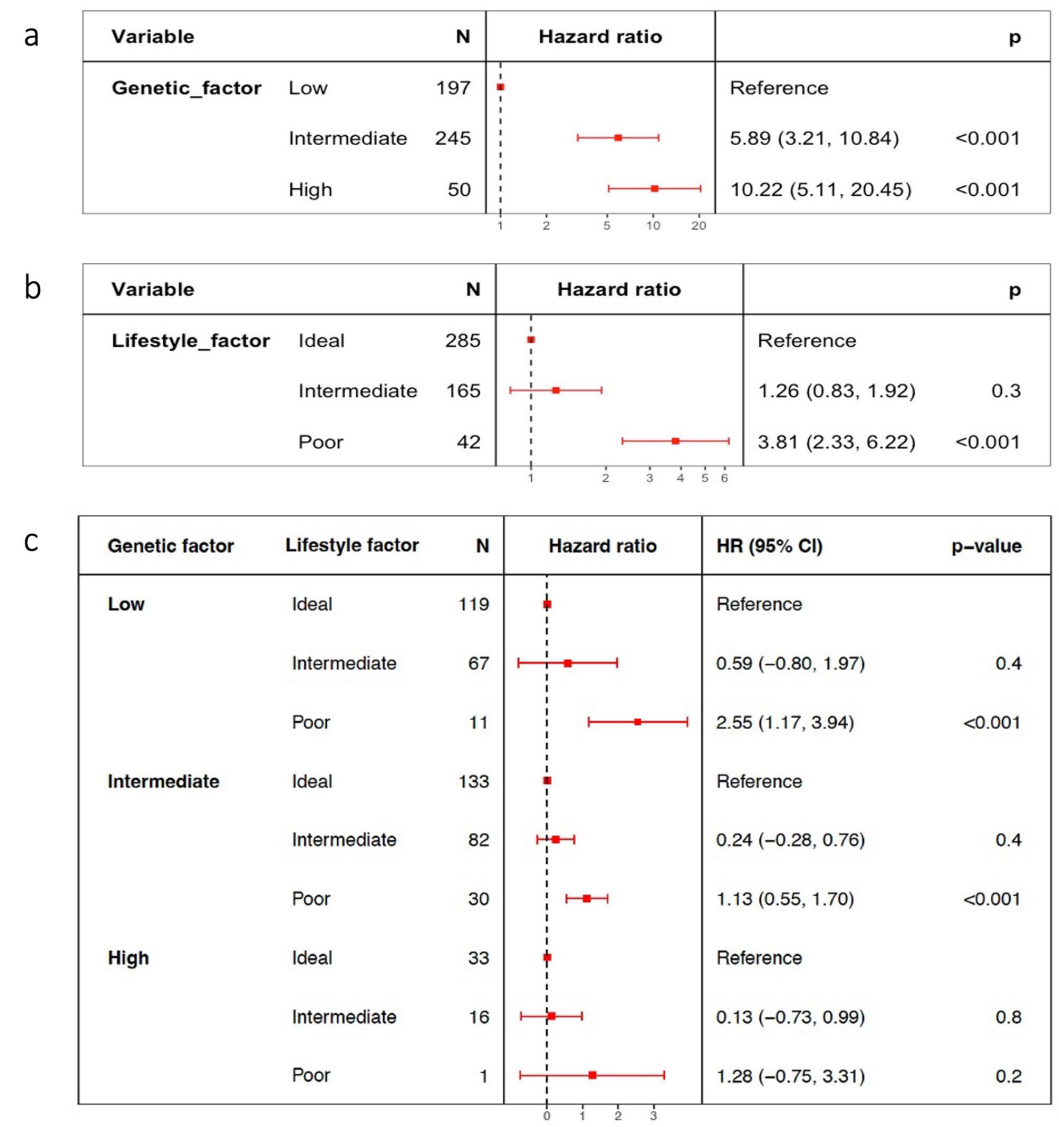
Risk of RCC according to genetic and lifestyle-associated factors. The risk of RCC was affected by genetic and lifestyle-associated factors. (**a**) Association of genetic factor with RCC risk. (**b**) Association of lifestyle-associated factors with RCC risk. (**c**) Association of lifestyle-associated factors with the risk of RCC across strata of PRS. HR, hazard ratio; CI, confidence interval; N, number; RCC, renal cell carcinoma; PRS, polygenic risk score; p, *p*-value

We categorized the combined lifestyle score as Ideal, Intermediate, and Poor and the PRS as Low, Intermediate, and High for 492 individuals. In the Cox proportional hazard model with combined lifestyle scores and RCC risk, the Poor lifestyle category (HR = 3.81, 95% CI: 2.33–6.22) involved a risk that was three times higher than that of the Ideal lifestyle category. A high genetic risk (PRS) was significantly associated with the RCC risk (HR = 10.22, 95% CI: 5.11–20.45). When lifestyle factors associated with the risk of RCC were stratified by PRS in the Cox proportional hazard model, the probability of RCC risk was higher in the poor lifestyle score category across PRS strata (Fig. 3).

## Discussion

### PRS model for predicting RCC risk in the Korean population

The recent advancements in sequencing techniques and development of novel data analysis methods have enabled the identification of disease-associated variants with increased accuracy and abundance, resulting in a more accurate PRS model. However, applying the same set of variants to the PRS model across different ethnic populations has resulted in several inaccuracies. In this prospective study, we identified 43 Korean-specific variants of RCC risk in a Korean population and constructed an optimal PRS model with 31 of the 43 variants, showing an AUC of 0.774. Although we used the Korean population dataset to avoid the inclusion of the different allele frequencies among various ancestries in our study, population substructure could affect the construction of a precise PRS model. Therefore, we performed PCA to explore whether population substructure affected the construction of our model; the results confirmed that our datasets were composed of the specific Korean population without any substructures.

Although RCC is a common tumor worldwide, only a few studies have been conducted on its prediction models. Scelo et al. identified seven new RCC risk loci and validated six known RCC risk loci by conducting a meta-analysis and performed PRS analysis on individuals of European ancestry. The authors focused on identifying rare variants for Europeans, which did not overlap with our Korean-specific variants [6]. To the best of our knowledge, this study is the first to construct a PRS model to predict the risk of RCC in the underrepresented Korean population.

### Non-coding DNA variants and biological mechanisms

Fifteen of the 31 Korean-specific variants identified in this study indirectly contribute to cancer initiation and progression. These intronic variants regulate genes such as enhancers, repressors, or promoters, and are involved in biological functions and pathways associated with the development of cancers by exerting oncogenic or tumor-suppressive effects in multiple organs [31]. Well-annotated pathways were related to the genes affected by the variants implicated in RCC. For example, the *RPTOR* gene, located in the 17q25.3 region, codes for a subunit of the mTORC1 complex, which is crucial for regulating various cellular processes, such as assembly, localization, and substrate binding of mTORC1. The PI3K/AKT/mTOR signaling pathway is an intracellular pathway that plays a vital role in cell cycle regulation, including the G0 phase and cell proliferation. PI3K, a lipid kinase, produces phosphatidylinositol-3,4,5-trisphosphate, a key second messenger that facilitates AKT translocation to the plasma membrane. AKT activation is central to fundamental cellular functions, such as cell proliferation and survival, as it phosphorylates various substrates. Dysregulation of this pathway is frequently observed in human cancers, particularly in RCC, and has been linked to aggressive tumor development and reduced survival rates [32–34]. The SUSD5 protein encoded by the *SUSD5* gene in the 3p22.3 region is expected to have hyaluronic acid-binding activity and play a role in the Notch signaling pathway. Notch signaling is crucial in regulating cell fate, proliferation, and death during development. It operates mainly between adjacent cells as its ligands are transmembrane proteins. Despite its simplicity in intracellular signaling with no secondary messengers, the Notch pathway is part of various developmental processes, and its dysfunction is implicated in many cancers, including RCC [35, 36].

### Relationship between lifestyle-associated factors and genetic risk expressed as PRS

Both lifestyle-associated factors and PRS were significantly associated with RCC risk, and lifestyle-associated factors affected RCC risk across PRS strata. However, Cox proportional hazard analysis showed no evidence that lifestyle-associated factors and PRS directly interacted with each other. Numerous studies have recently reported the relationship between epigenetic markers and lifestyle-associated factors, such as stress, smoking, alcohol use, and diet [37]. Various environmental factors epigenetically remodel the genome without altering its DNA sequence. Epigenetic markers influence the modulation of gene expression and thus play a critical role in health status and prevention of cancers and complex diseases [38].

The last 15 of the 31 Korean-specific variants identified in this study were intergenic variants. Many intergenic variants can affect gene regulation through epigenetic modifications, such as chromatin remodeling or histone modifications, including methylation or acetylation. Modulated expression of oncogenes and tumor suppressor genes affects cancer development [39]. In the present study, among the 15 intergenic variants, rs73149350 is situated in an open chromatin region of the genome. The open chromatin region is accessible and has a less condensed chromatin structure, facilitating the binding of transcription factors and other regulatory proteins to the DNA. The *SEMA3C* gene, in closest proximity to rs73149350, contributes to the promotion of cancer cell growth [40]. Therefore, rs73149350 may potentially regulate *SEMA3C* expression through processes such as chromatin remodeling or histone modification. This regulatory effect could have implications for the risk associated with RCC. However, it is important to note that further studies are needed to fully understand the biological mechanisms underlying the regulation of genes by these intergenic variants. The finding suggest that lifestyle-associated factors may indirectly affect acquired risk factors through epigenetic modulation [41].

### Limitations and future directions

This study has certain limitations. First, we did not perform additional pathway or biological mechanism analysis of the intergenic variants. Without these analyses, the biological relevance of these variants in the context of RCC risk may remain unclear. Second, epigenetic association studies should be conducted to draw more accurate inferences. We must investigate the specific epigenetic mechanisms through which lifestyle-associated factors, such as stress, smoking, alcohol use, and diet, influence gene expression and how these modifications are related to RCC risk. This investigation could involve detailed epigenome-wide association studies to identify specific epigenetic changes associated with lifestyle factors. Further in-depth studies are required to explore the relationship between lifestyle-associated factors and genetic risk. These studies should consider incorporating such analyses to gain a deeper understanding of the underlying biology and potentially develop clinical applications.

## Conclusion

The aim of the present study was to construct a Korean-specific PRS model that predicts the risk of RCC development and to explore the association of lifestyle-associated factors with the genetic factor influencing RCC risk. To mitigate the impact of ethnicity, GWAS analysis was exclusively performed on the underrepresented Korean population, leading to the identification of Korean-specific variants associated with RCC risk. The Korean-specific PRS model was constructed with 31 identified variants and demonstrated a robust prediction rate of 77.4%. Among the 31 variants, 15 intronic variants indirectly contributed to cancer initiation and progression through their involvement in key biological functions and pathways such as PI3K/AKT/mTOR or Notch signaling pathway. The remaining 15 intergenic variants potentially impact gene regulation through epigenetic modifications such as methylation or histone modification. Epigenetic modification is known to be influenced by environmental factors including lifestyle-associated factors. Furthermore, we investigated the association between lifestyle-associated factors, such as physical activity, alcohol use, smoking habit, and diet, and the risk of RCC development. Our results suggest that lifestyle-associated factors may indirectly influence acquired risk factors through epigenetic modification. However, further studies that delve deeper into these complex interactions and facilitate a comprehensive understanding of the interplay between genetic factors and lifestyle-associated factors in relation to RCC risk are warranted.

### Electronic supplementary material

Below is the link to the electronic supplementary material.

Additional File 1

## Data Availability

All data used in this study are available at the National Biobank of Korea website (https://biobank.nih.go.kr/cmm/main/mainPage.do) and the Seoul National University Prospectively Enrolled Registry for Genitourinary Cancer (SUPER-GUC). The SUPER-GUC is available at 10.4111/icu.2019.60.4.235.

## Abbreviations

AUC: Area under the curve
BMI: Body mass index
FDR: False discovery rate
GWAS: Genome-wide association studies
HWE: Hardy–Weinberg Equilibrium
KoGES: Korean Genome and Epidemiology Study
MAF: Minor allele frequency
PCA: Principal component analysis
PRS: Polygenic risk score
QC: Quality control
QQ-plot: Quantile–quantile plot
RCC: Renal cell carcinoma
ROC: Receiver operating characteristic
SNP: Single nucleotide polymorphism
SUPER-RCC-Nx: Seoul National University Prospectively Enrolled Registry for RCC-Nephrectomy
