# Supplementary material for: Polygenic risk score model for renal cell carcinoma in the Korean population and relationship with lifestyle-associated factors
Source: BMC Genomics. 2024 Jan 10;25:46. doi: 10.1186/s12864-024-09974-w (PMC10777500; doi:10.1186/s12864-024-09974-w)
Supplement: Supplementary file 1 — Additional File 1 [file 12864_2024_9974_MOESM1_ESM.docx]

Additional File 1

Polygenic risk score model for renal cell carcinoma in the Korean population and relationship with lifestyle-associated factors

**Joo Young Hong, Jang Hee Han, Seung Hwan Jeong, Cheol Kwak, Hyeon Hoe Kim, Chang Wook Jeong***

# Supplementary Figures


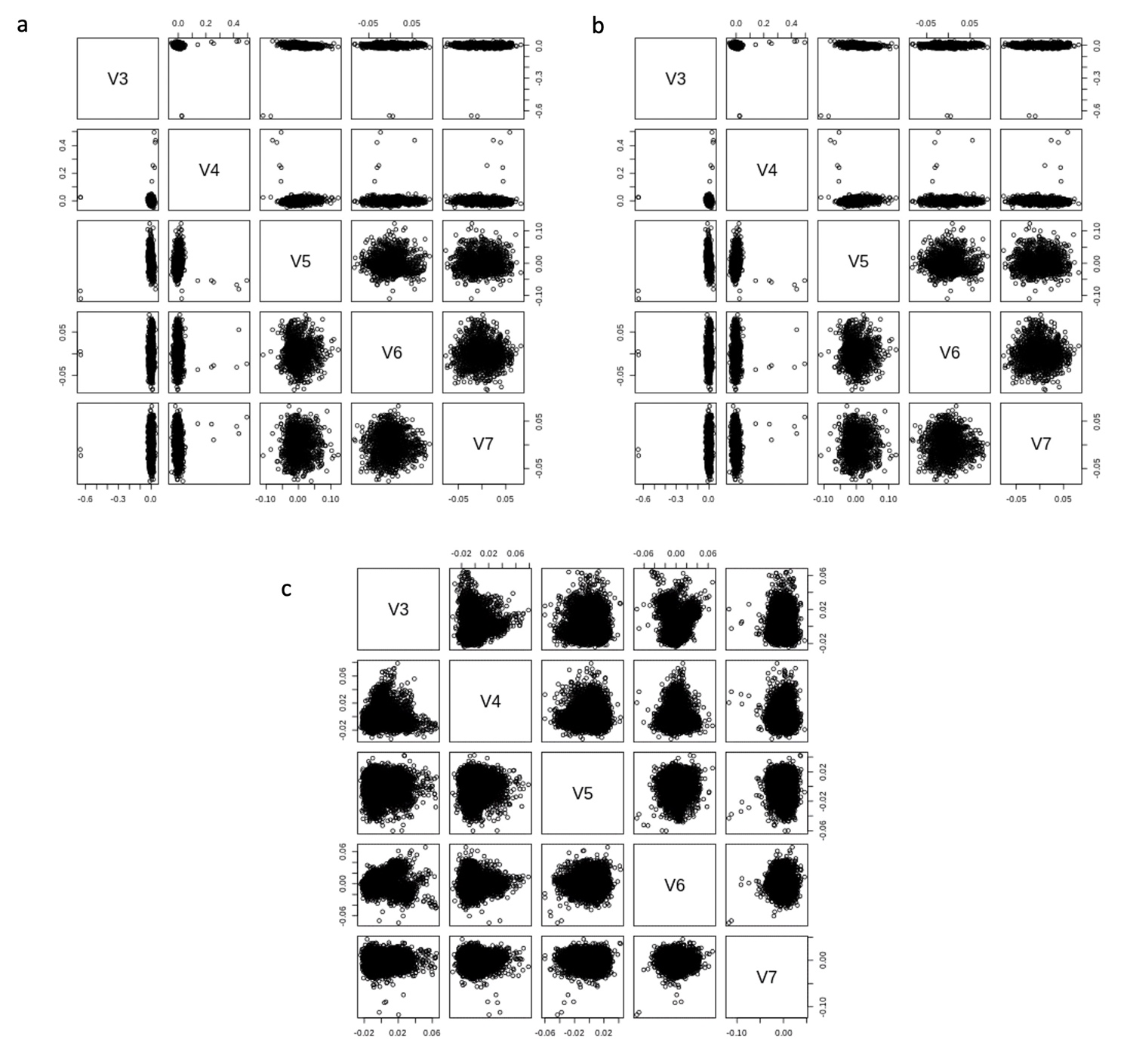


**Figure S1.** Biplots of PCA. (a) Biplots of PCA for cases before merging (after batch effect correction): 1,104 samples. (b) Biplots of PCA for controls before merging (after batch effect correction): 3,811 samples. (c) Biplots of PCA for cases and controls after merging and QC: 4,915 samples. PCA, principal component analysis.


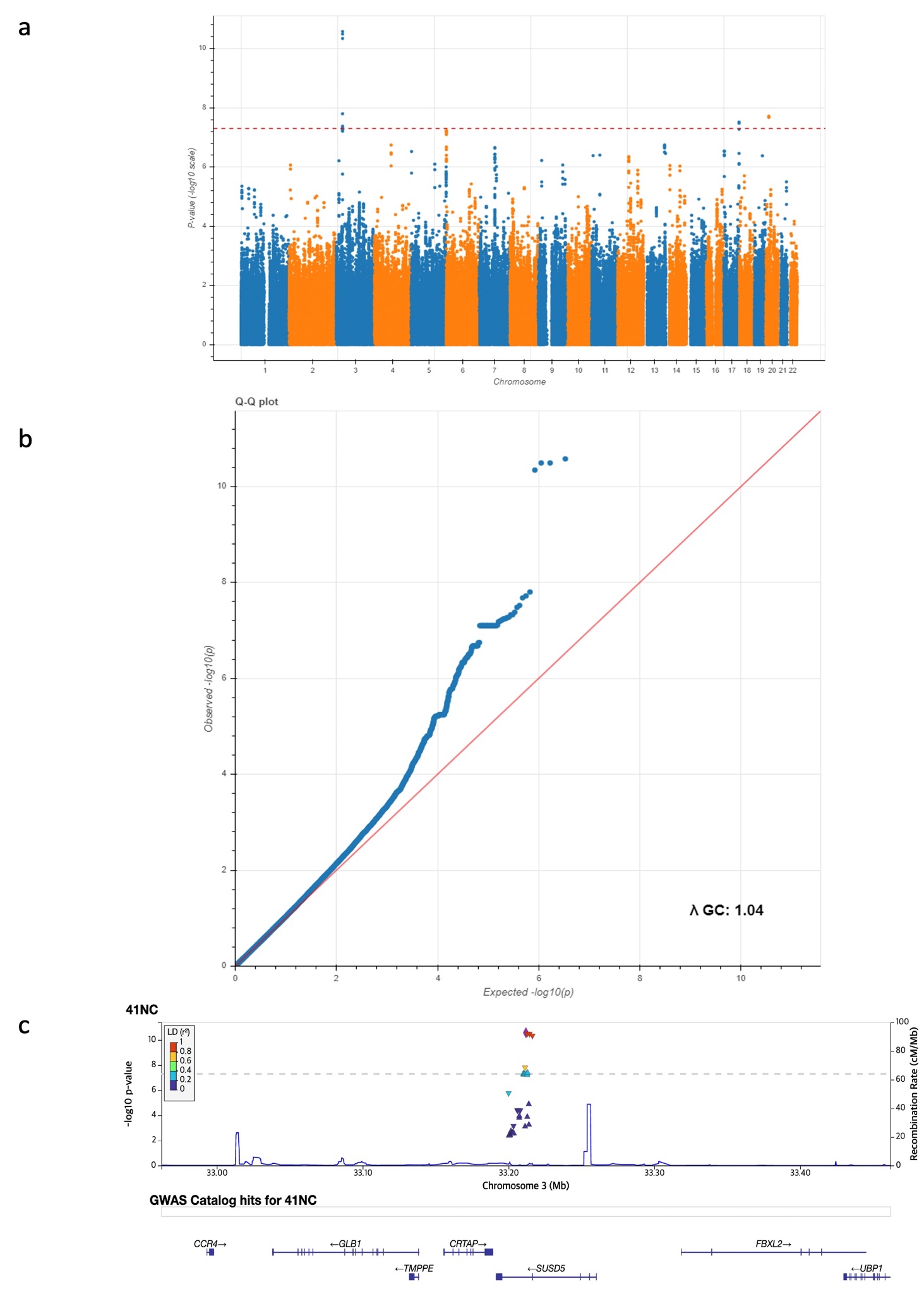


**Figure S2.** Manhattan plot, QQ-plot, and LocusZoom plot. (a) Manhattan plot. The Manhattan plot is a scatter plot of the association of the negative logarithm of the *p*-value with the chromosomal location. It presents genome-wide SNPs significantly associated with RCC. (b) QQ-plot. The QQ-plot presents the deviation of the observed *p*-value versus the expected *p*-value from the null hypothesis based on the lambda statistic λ, a measure of the inflated *p*-value. Here, λ = 1.04. (c) LocusZoom plot. The LocusZoom plot shows that rs67756935 is a significant SNP in the *SUSD5* gene. RCC, renal cell carcinoma; QQ-plot, quantile–quantile plot; λ, lambda.


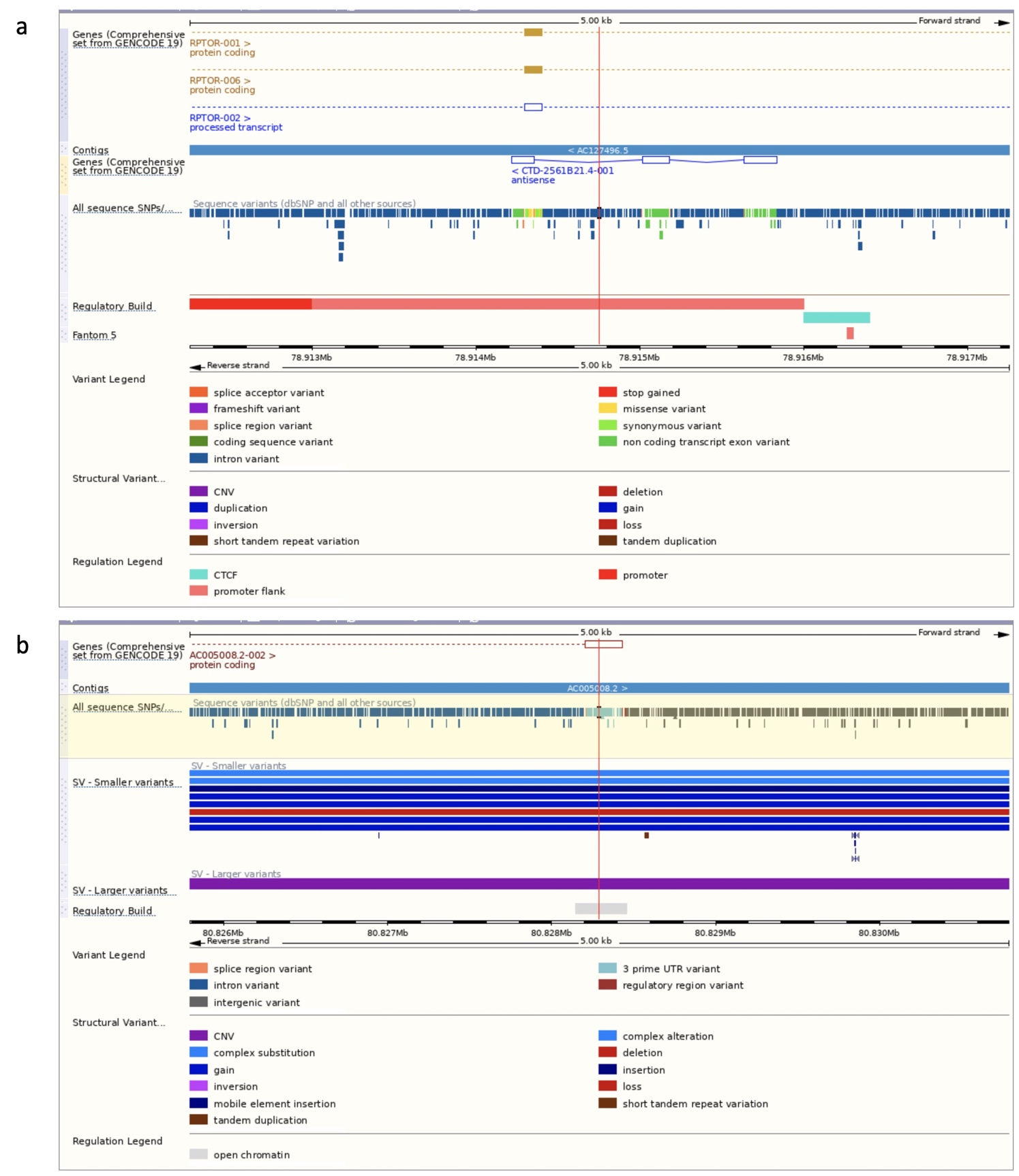


**Figure S3.** SNPs of gene variants. (a) rs908237 intronic variant in the *RPTOR* gene. (b) rs73149350 intergenic (3 prime UTR) variant near the *SEMA3* gene.

# Supplementary Tables

**Table S1.** Characteristics of favorable lifestyle-associated factors at baseline (*n*=492).

| Lifestyle-associated factors | Favorable | Unfavorable |
| --- | --- | --- |
| BMI <30 kg/m^2^ | 463 | 29 |
| No smoking | 271 | 221 |
| Moderate alcohol intake | 261 | 231 |
| No history of hypertension | 350 | 142 |

**Table S2.** SNPs significantly associated with RCC in the Korean population (*n*=43).

| rsID | CHR | POS | REF | ALT | AF | *P* | OR |
| --- | --- | --- | --- | --- | --- | --- | --- |
| rs72851444 | 1 | 4203990 | G | A | 0.0662 | 4.41E-06 | 5.46E-01 |
| rs12135478 | 1 | 39539043 | A | G | 0.07556 | 5.25E-06 | 5.83E-01 |
| rs57315464 | 1 | 69152589 | G | T | 0.06635 | 5.87E-06 | 5.57E-01 |
| rs1108459 | 2 | 5674344 | G | T | 0.09747 | 5.96E-06 | 5.95E-01 |
| rs56354798 | 2 | 6594126 | T | A | 0.08099 | 8.53E-07 | 5.49E-01 |
| rs76670552 | 2 | 142213267 | G | T | 0.09016 | 9.49E-06 | 6.03E-01 |
| rs35005811 | 3 | 14376664 | T | G | 0.24072 | 6.14E-07 | 1.41E+00 |
| rs67756935 | 3 | 33211952 | C | T | 0.0958 | 2.66E-11 | 4.36E-01 |
| rs3732401 | 3 | 120500093 | C | T | 0.48918 | 7.00E-06 | 1.31E+00 |
| rs76221875 | 4 | 86702592 | G | A | 0.09587 | 1.81E-07 | 5.53E-01 |
| rs4635969 | 5 | 1308552 | G | A | 0.09294 | 2.98E-07 | 5.52E-01 |
| rs78544457 | 5 | 121659085 | G | T | 0.10057 | 7.96E-07 | 5.84E-01 |
| rs17719136 | 5 | 147578177 | G | C | 0.08744 | 4.39E-06 | 5.88E-01 |
| rs79192363 | 5 | 180458802 | G | A | 0.07741 | 9.88E-07 | 5.29E-01 |
| rs6597341 | 6 | 914841 | C | T | 0.09013 | 5.83E-08 | 5.34E-01 |
| rs75659100 | 6 | 120041218 | A | G | 0.0844 | 5.73E-06 | 5.82E-01 |
| rs4897331 | 6 | 129866547 | C | T | 0.08752 | 3.71E-06 | 5.80E-01 |
| rs73149350 | 7 | 80828288 | G | A | 0.0721 | 2.19E-07 | 5.01E-01 |
| rs75995217 | 7 | 88849880 | C | G | 0.07559 | 9.60E-07 | 5.41E-01 |
| rs56129741 | 7 | 151511382 | C | G | 0.09616 | 7.65E-06 | 6.04E-01 |
| rs78392740 | 8 | 74286584 | A | G | 0.10749 | 4.91E-06 | 1.54E+00 |
| rs57845130 | 9 | 17854783 | G | T | 0.07597 | 5.99E-07 | 5.23E-01 |
| rs73584329 | 9 | 127047016 | C | T | 0.09449 | 8.12E-07 | 5.68E-01 |
| rs74588014 | 9 | 138726580 | C | A | 0.0938 | 2.64E-06 | 5.85E-01 |
| rs11041484 | 11 | 7641520 | G | A | 0.08508 | 4.12E-07 | 5.45E-01 |
| rs117352285 | 11 | 42410962 | C | T | 0.08522 | 3.94E-07 | 5.34E-01 |
| rs11829786 | 12 | 57332232 | C | T | 0.07762 | 4.43E-07 | 5.28E-01 |
| rs7973376 | 12 | 66918796 | T | G | 0.28683 | 3.50E-06 | 7.32E-01 |
| rs12813302 | 12 | 103856908 | G | A | 0.09099 | 1.28E-06 | 5.64E-01 |
| rs200334091 | 13 | 108890247 | C | T | 0.0891 | 1.79E-07 | 5.54E-01 |
| rs72661905 | 13 | 113568986 | C | T | 0.07805 | 3.46E-07 | 5.28E-01 |
| rs116907254 | 14 | 21313268 | C | T | 0.09977 | 8.88E-07 | 5.77E-01 |
| rs12050132 | 14 | 73276250 | A | T | 0.50742 | 9.27E-07 | 1.34E+00 |
| rs11624534 | 14 | 97578977 | G | C | 0.25655 | 9.64E-06 | 1.34E+00 |
| rs13333942 | 16 | 81579616 | A | G | 0.24985 | 5.62E-06 | 1.35E+00 |
| rs11868975 | 17 | 2664626 | A | G | 0.09587 | 2.85E-07 | 5.49E-01 |
| rs2242402 | 17 | 77918261 | G | A | 0.09121 | 4.25E-06 | 5.93E-01 |
| rs908237 | 17 | 78914751 | G | A | 0.10847 | 3.01E-08 | 5.42E-01 |
| rs13381896 | 18 | 25356190 | T | G | 0.90662 | 1.97E-06 | 6.31E-01 |
| rs75488411 | 19 | 41012097 | C | T | 0.09048 | 4.17E-07 | 5.46E-01 |
| rs6110518 | 20 | 15085205 | G | T | 0.08761 | 1.92E-08 | 5.04E-01 |
| rs6138460 | 20 | 24979778 | C | G | 0.07692 | 5.77E-06 | 5.70E-01 |
| rs434919 | 21 | 43333005 | G | T | 0.07181 | 3.14E-06 | 5.33E-01 |
